# *Plasmodium falciparum* Liver Stage Infection and Transition to Stable Blood Stage Infection in Liver-Humanized and Blood-Humanized FRGN KO Mice Enables Testing of Blood Stage Inhibitory Antibodies (Reticulocyte-Binding Protein Homolog 5) *In Vivo*

**DOI:** 10.3389/fimmu.2018.00524

**Published:** 2018-03-14

**Authors:** Lander Foquet, Carola Schafer, Nana K. Minkah, Daniel G. W. Alanine, Erika L. Flannery, Ryan W. J. Steel, Brandon K. Sack, Nelly Camargo, Matthew Fishbaugher, Will Betz, Thao Nguyen, Zachary P. Billman, Elizabeth M. Wilson, John Bial, Sean C. Murphy, Simon J. Draper, Sebastian A. Mikolajczak, Stefan H. I. Kappe

**Affiliations:** ^1^Center for Infectious Disease Research, Seattle, WA, United States; ^2^Jenner Institute, University of Oxford, Oxford, United Kingdom; ^3^Department of Laboratory Medicine, University of Washington, Seattle, WA, United States; ^4^Department of Microbiology, University of Washington, Seattle, WA, United States; ^5^Yecuris Corporation, Tualatin, OR, United States; ^6^Department of Global Health, University of Washington, Seattle, WA, United States

**Keywords:** *Plasmodium falciparum*, humanized mouse model, *Plasmodium falciparum* blood stages, reticulocyte-binding protein homolog 5, clodronate liposomes, cyclophosphamide

## Abstract

The invention of liver-humanized mouse models has made it possible to directly study the preerythrocytic stages of *Plasmodium falciparum*. In contrast, the current models to directly study blood stage infection *in vivo* are extremely limited. Humanization of the mouse blood stream is achievable by frequent injections of human red blood cells (hRBCs) and is currently the only system with which to study human malaria blood stage infections in a small animal model. Infections have been primarily achieved by direct injection of *P. falciparum*-infected RBCs but as such, this modality of infection does not model the natural route of infection by mosquito bite and lacks the transition of parasites from liver stage infection to blood stage infection. Including these life cycle transition points in a small animal model is of relevance for testing therapeutic interventions. To this end, we used FRGN KO mice that were engrafted with human hepatocytes and performed a blood exchange under immune modulation to engraft the animals with more than 50% hRBCs. These mice were infected by mosquito bite with sporozoite stages of a luciferase-expressing *P. falciparum* parasite, resulting in noninvasively measurable liver stage burden by *in vivo* bioluminescent imaging (IVIS) at days 5–7 postinfection. Transition to blood stage infection was observed by IVIS from day 8 onward and then blood stage parasitemia increased with a kinetic similar to that observed in controlled human malaria infection. To assess the utility of this model, we tested whether a monoclonal antibody targeting the erythrocyte invasion ligand reticulocyte-binding protein homolog 5 (with known growth inhibitory activity *in vitro*) was capable of blocking blood stage infection *in vivo* when parasites emerge from the liver and found it highly effective. Together, these results show that a combined liver-humanized and blood-humanized FRGN mouse model infected with luciferase-expressing *P. falciparum* will be a useful tool to study *P. falciparum* preerythrocytic and erythrocytic stages and enables the testing of interventions that target either one or both stages of parasite infection.

## Introduction

More than 200 million clinical cases of malaria are reported each year, with children under the age of 5 being particularly susceptible to illness and death. *Plasmodium falciparum* is the most lethal human malaria parasite (WHO World Malaria Report 2016) and continued discovery and development of interventions against it is necessitated by the occurrence of drug resistance and the lack of an effective vaccine. Transmission of *Plasmodium* parasites occurs by the bite of infected female *Anopheles* mosquitoes, which inject motile sporozoites into the skin where they traverse endothelial cells to enter the bloodstream and travel to the liver. In the liver, sporozoites infect hepatocytes, which marks the beginning of the asymptomatic liver stage infection. Within a time period of 6–7 days, parasites mature inside hepatocytes and eventually form tens of thousands of merozoites, which are released into the bloodstream where they invade red blood cells (RBCs). Blood stage infection becomes symptomatic and the cyclic infection and destruction of RBCs by the parasite as well as the adhesion of infected RBCs to the vascular endothelium, causes the morbidity and mortality associated with infection.

Although *P. falciparum* can be cultured *in vitro*, several aspects of malaria infections can only be addressed by *in vivo* research. The complex mechanisms of malaria transmission through mosquito bites, the multiple tissue barriers crossed by the parasite, and the different cell types that are infected during its life cycle make it impossible to study all aspects in one *in vitro* system. Also, drug studies will require an *in vivo* system for PK/PD analysis and for prodrugs, which will not be metabolized *in vitro* and therefore their potential antimalarial activity cannot be assessed (1). The standardization of controlled human malaria infections (CHMI) has made it possible to study the efficacy of novel drugs and vaccines in the human system (2). However, the high cost and ethical considerations involved with CHMI necessitate the testing of new compounds in relevant animal models prior to moving them forward in clinical trials.

The recent development of liver-humanized mouse models has made it possible to study *P. falciparum* liver stage infection *in vivo*. One of these mouse models is the FRG KO mouse which was developed by adding Rag2^−/−^ and IL2rg^−/−^ immunodeficiency backgrounds to the C57BL/6 fumarylacetoacetate hydrolase (FAH) knock-out mouse developed by Grompe et al. (3). The FAH KO results in a defect in the tyrosine catabolic pathway, which leads to the accumulation of maleylacetoacetate and fumarylacetoacetate, upstream of the FAH blockade. These metabolites are highly reactive and unstable, and upon breakdown cause hepatocellular injury (3). This toxicity can be prevented by oral administration of 2-(2-nitro-4-trifluoro-methylbenzoyl)-1,3-cyclohexanedione (NTBC), which blocks the tyrosine catabolism pathway upstream of the toxic metabolites (4). Liver injury can be induced by withdrawing mice from NTBC, thereby allowing repopulation of the diseased mouse liver with human hepatocytes (5).

The Rag2^−/−^ and IL2rg^−/−^ mutations prevent the development of B cells, T cells, and NK cells. Backcrossing of the FRG mouse onto the non-obese diabetic (NOD) background enables additional repopulation of the resulting NOD Fah^−/−^Rag2^−/−^IL2rg^−/−^ (FRGN KO) mice with hematopoietic stem cells (HSCs). The NOD background has various intrinsic immune deficiencies, most importantly, it carries a polymorphism in the SIRPα gene that improves crosstalk between CD47 on human cells and the SIRPα receptor on mouse phagocytes, thereby preventing NOD macrophages from engulfing human grafts (6).

The transplantation of immunodeficient mice with CD34 + HSCs leads to the development of most human blood cell lineages. Unfortunately, human erythropoiesis is not sufficient in these mice, therefore, only very low amounts of human red blood cells (hRBCs) can be detected in the periphery (7–9). The only current method to achieve high amounts of hRBCs in mice is the frequent injection of large volumes of hRBCs. One option to study *P. falciparum* blood stages *in vivo* is the injection of *in vitro* cultured asexual stage parasites into immunodeficient mice that have been preloaded with hRBCs (10, 11). By combining this approach with the injection of macrophage and neutrophil-depleting chemicals, the development of gametocytes that sequester in spleen and bone marrow could be observed, therefore somewhat mimicking human infection (12). A disadvantage of this system is that it does not model the natural route of infection by mosquito bite and it lacks liver to blood stage transition. These life cycle transition stages are important to include in a mouse system that will serve as a model for the complete *P. falciparum* life cycle. Especially the transition from liver to blood stage is a critical step in the parasite life cycle and provides a target for intervention with drugs and vaccines in order to prevent the establishment of a blood stage infection. Therefore, it should be included in a mouse model for malaria drug and vaccine testing.

Liver to blood stage transition has been reported previously in the liver humanized TK-NOG mouse. Similar to other NSG models, the blood stream of these mice can be reconstituted with hRBCs by daily intraperitoneal (i.p.) injections of 1 ml hRBCs starting 6 days before i.v. injection of a large number of *P. falciparum* sporozoites, thereby allowing liver stage to blood stage transition and subsequent blood stage infection. The numbers of sexual and asexual stages were highly variable in these mice and *in vivo* solely detected by thin blood smears (13). The liver humanized FRGN KO (FRGN huHep) mouse can also be reconstituted with human erythrocytes, thus allowing the transition of *P. falciparum* liver to blood stage infection, which can be further propagated in an *in vitro* culture after exsanguinating the mice (14).

Here, we report the development of a protocol for the long-term engraftment of hRBCs in *P. falciparum* sporozoite-infected FRGN huHep mice. As mosquito bite is the natural route of infection, we have included this modality of *P. falciparum* sporozoite transmission. Following our protocol for hRBC engraftment, we achieve liver to blood stage transition followed by the stable maintenance of a *P. falciparum* blood stage infection with increasing parasitemia. The transition from liver stage to blood stage is visualized by *in vivo* bioluminescent imaging (IVIS), allowing the discrimination of treatment efficacy against either one or both stages. We assessed the utility of this model by showing that blood stage infection emerging from the liver can be blocked successfully by a monoclonal antibody (mAb) recognizing *P. falciparum* reticulocyte-binding protein homolog 5 (RH5). RH5 is an essential merozoite invasion ligand that interacts with the basigin receptor on hRBCs. This interaction is a prerequisite for infection throughout all parasite strains tested to date (15, 16). Recently, the first clinical study assessing RH5 as a vaccine candidate was conducted. Substantial RH5-specific immune responses could be induced by immunization of malaria-naïve individuals with viral vectors encoding PfRH5 (17). The RH5–basigin interaction therefore constitutes an important potential vaccine target. The inhibition of *P. falciparum* blood stage infection by an anti-RH5 mAb in our mouse model underlines its utility for the study of potential antibody-based malaria intervention strategies.

## Results and Discussion

*Plasmodium falciparum* liver infection can be studied *in vivo* using liver humanized FRG KO or FRGN KO mice. Infection with sporozoites of luciferase-expressing parasites made it possible to follow the progression of infection by *in vivo* bioluminescent imaging (IVIS), without sacrificing the animal. Liver infection was detected until day 7 postinfection. One injection of hRBCs on day 6 postinfection allowed parasites to transition from liver stage to blood stage infection. The parasitemia was detectable by quantitative PCR and the infection can be propagated *in vitro* after exsanguinating the mice (14). Unfortunately, this method does not allow a stable blood stage infection *in vivo* due to rapid clearance of infected hRBCs (iRBC) by mouse phagocytes.

Here, we addressed this issue and developed an immune modulation protocol that allows the iRBCs to remain in the circulation without being phagocytosed. The efficient engraftment of sporozoite infected mice with high amounts of hRBCs leads to expansion of the infection and thereby increasing parasitemia over time.

## Development of an Immune Modulation Protocol to Prevent Phagocytosis of iRBCs

To prevent phagocytosis of iRBCs, we optimized an immune modulation protocol to specifically eliminate mouse phagocytes, namely macrophages and neutrophils. As previously described, mouse macrophages can be eliminated by the administration of clodronate-containing liposomes, which are phagocytosed by macrophages and subsequently induce apoptosis (18). Experimental neutropenia is commonly induced either by administration of antibodies targeting neutrophil-specific receptors such as Ly6G (19) or by the cytotoxic chemotherapy agent cyclophosphamide (20). Compared to antibodies, which are highly specific, cyclophosphamide has a broader immunosuppressive effect, as it targets all dividing cells. Therefore, we decided to utilize this method to deplete neutrophils, as any further immune suppression could aid in preventing phagocytosis of iRBCs.

**Figure 1:**
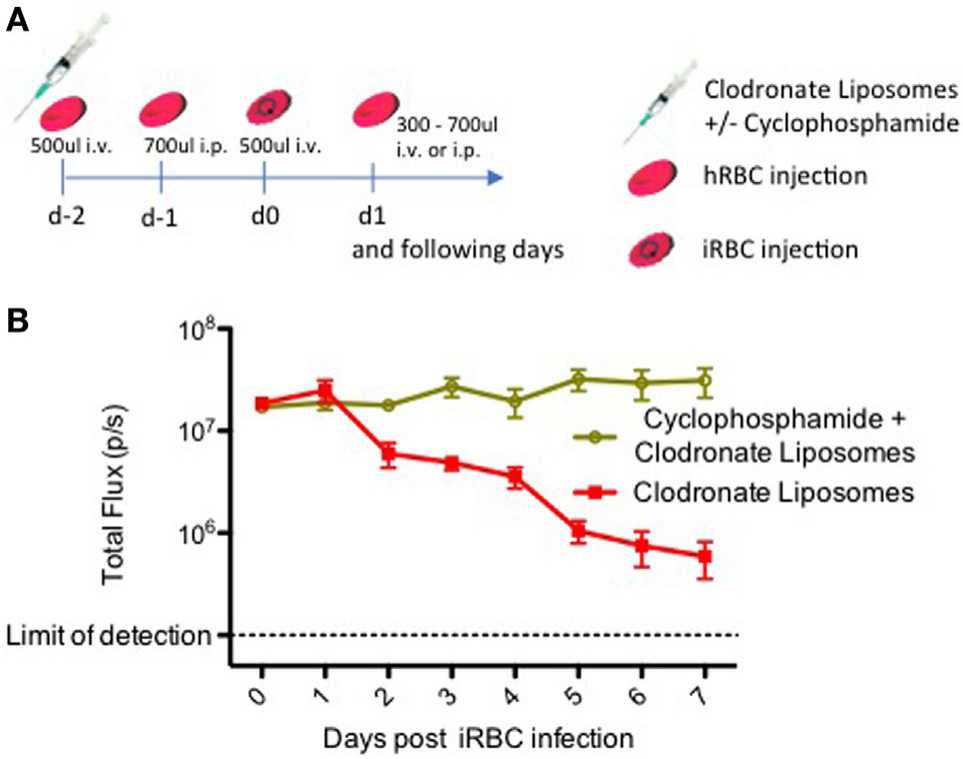
Immune modulation with clodronate liposomes and cyclophosphamide leads to a stable *Plasmodium falciparum* blood stage infection. **(A)** Timeline showing the protocol for the repopulation of liver-humanized FRGN KO mice with human red blood cells (hRBCs) and subsequent infection with blood stage parasites. This protocol was utilized here to assess the effect of cyclophosphamide on a *P. falciparum* blood stage infection in FRGN huHep mice. Six mice received an injection of 50 µl CloLip i.v. + 50 µl CloLip i.p. on day −2 and three mice additionally received 150 mg/kg (approximately 150 µl per mouse) cyclophosphamide i.p. All animals were bled 200 µl, and received one i.v. injection of 500 µl hRBCs. On day −1, the animals were bled 100 µl and received an i.p. injection of 700 µl hRBCs. On day 0, the animals were bled 100 µl and received an i.v. injection of 500 µl hRBCs containing 1 × 10^7^ iRBCs. The following days, all mice received an individually determined amount of hRBCs. Parasitemia was followed by daily intravital imaging **(B)**. The IVIS signal of mice treated only with CloLip started decreasing on day 2 postinfection. Only the mice which received both CloLip and cyclophosphamide, showed a stable *P. falciparum* blood stage infection throughout the 7-day observation period.

To assess the effect of clodronate-containing liposomes (CloLip; Clophosome^®^-A, FormuMax) alone and in combination with cyclophosphamide (Sigma Aldrich, St. Louis, MO, USA), six liver humanized FRGN KO mice received an injection of 50 µl CloLip i.v. + 50 µl CloLip i.p. on day −2 before infection to remove macrophages and monocytes from both the circulation and the peritoneal cavity. Three mice additionally received 150 mg/kg cyclophosphamide i.p. All animals were bled 200 µl, and received an i.v. injection of 500 µl hRBCs [70% O + human erythrocytes in RPMI 1640 (25 mM HEPES, 2 mM l-glutamine) supplemented with 50 µM hypoxanthine plus 10% human serum and 5 µl penicillin–streptomycin (Gibco™, 10,000 U/ml penicillin, 10,000 µg/ml streptomycin)] to preload the mice with a pool of hRBCs. On day −1, the animals were bled 100 µl and received an i.p. injection of 700 µl hRBCs. On day 0, the animals were bled 100 µl and received an i.v. injection of 500 µl hRBCs containing 1 × 10^7^ *P. falciparum* NF54 GFP-Luc iRBCs from an *in vitro* blood culture. On the day of infection, the animals were reconstituted with approximately 20–30% hRBCs independent of the immune modulation protocol. The animals received an individually determined amount of hRBCs each day to stabilize the percentage between 50 and 70%. This protocol is depicted in Figure 1A.

Our results in Figure 1B show that elimination of monocytes with CloLip is beneficial, but not yet sufficient to enable a stable blood stage infection, as the parasitemia starts to decline as early as 2 days postinfection. Only the additional elimination of neutrophils with cyclophosphamide results in a stable blood stage infection quantifiable by IVIS (Figure 1B). In contrast to mice treated with CloLip alone, the IVIS signal of macrophage and neutrophil depleted mice remained stable over the 7-day observation period, indicating that the iRBCs are not being cleared. Notably, this immunomodulation protocol did not lead to any loss of mice throughout the experiment.

## Repopulation of Sporozoite-Infected FRGN huHEP Mice with hRBCs

The immune modulation protocol described above had to be refined for the setting of mice infected with sporozoites by mosquito bite, as it is known that *Plasmodium* skin and liver stages induce an innate immune response in the host (21). In order to reduce this initial immune response, on the day of mosquito bite challenge mice were injected both in the retro-orbital plexus and the peritoneal cavity with 50 µl CloLip and 150 mg/kg cyclophosphamide i.p. The CloLip (50 µl i.v. and i.p.) and cyclophosphamide (following doses are 100 mg/kg i.p.) injections were repeated on days 5, 9, 11, and 13 postinfection, thereby preventing the iRBCs from being cleared from the circulation.

**Figure 2:**
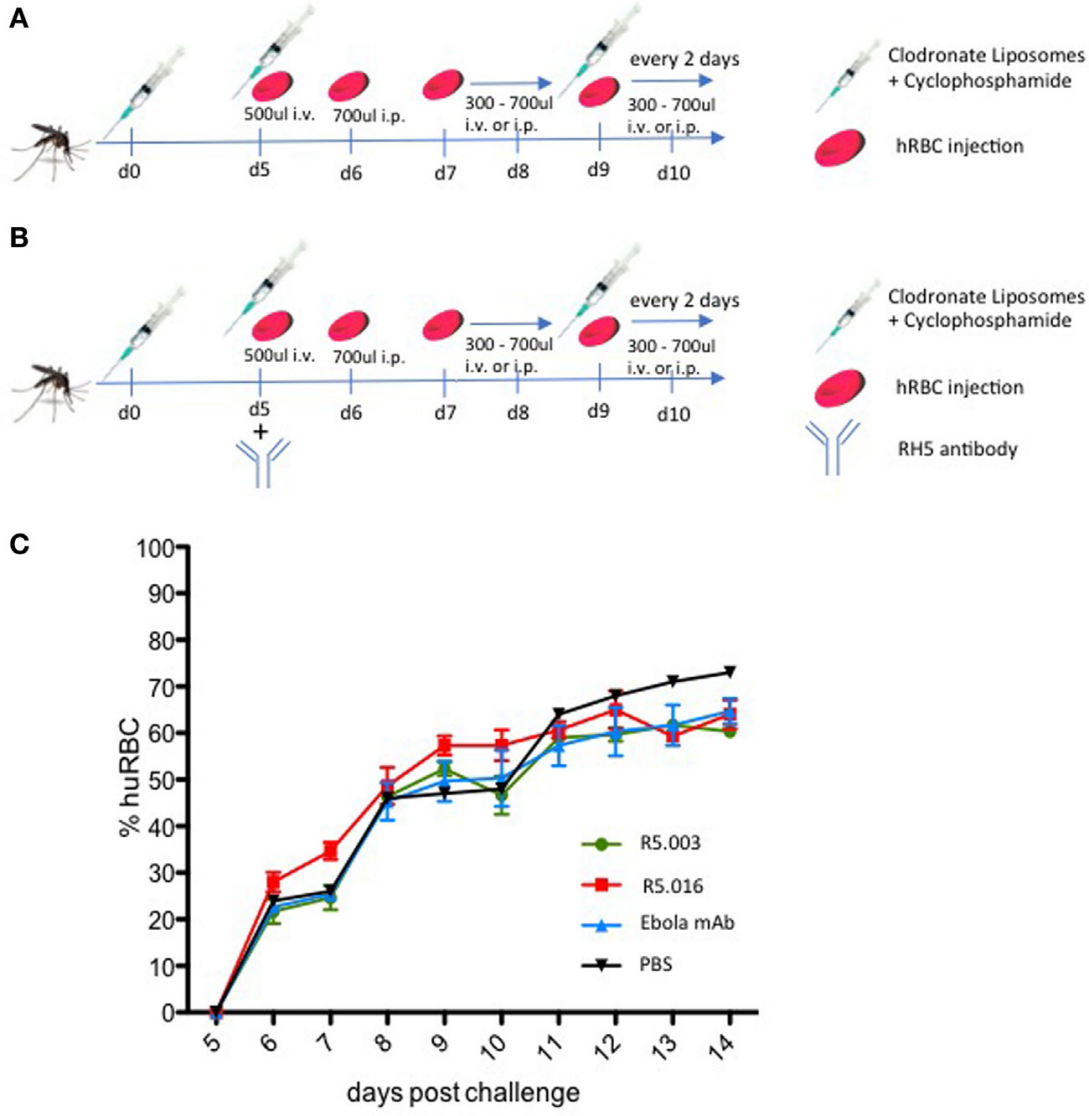
Protocol for the repopulation of sporozoite-infected FRGN mice with human red blood cells (hRBCs). **(A)** Mice are infected by mosquito bite with *Plasmodium falciparum* NF54 GFP-Luc sporozoites (*n* = 50 mosquitoes per mouse; 20 min). On the day of infection, mice are injected with 50 µl CloLip i.v. + 50 µl CloLip intraperitoneal (i.p.) and 150 mg/kg cyclophosphamide i.p. These injections are repeated on days 5, 9, 11, and 13 postinfection. On day 5 postinfection, 200 µl blood is drawn and the mice receive one injection of 500 µl hRBCs i.v. On day 6 postinfection, 100 µl blood is drawn and 700 µl hRBCs are injected i.p. The volumes of hRBCs injected the following days are determined individually in order to stabilize the percentages of hRBCs between 50 and 70%. **(B)** In order to assess the effect of antireticulocyte-binding protein homolog 5 antibody on transition and maintenance of a *P. falciparum* blood stage infection, blood humanization was achieved as described in **(A)**. On day 5 postmosquito-bite infection, all mice received one i.p. injection of 100 mg/kg of R5.003, R5.016, or control anti-Ebola monoclonal antibody (mAb) (EBL040). All three mAbs were human IgG1. One mouse received PBS as a control. **(C)** The percentages of hRBCs were assessed by FACS analysis using a CD235ab antibody. All mice show similar percentages of hRBCs throughout the 14-day observation period, independent of the treatment regimen, demonstrating that the differences observed in blood stage infection were not due to varying amounts of hRBCs. The mean ± SD is shown for each treatment group.

In order to rapidly repopulate mice with high amounts of hRBCs using a limited amount of injections, we optimized a blood exchange method where blood was drawn from the animals daily during the final days of the liver stage development while concurrently receiving large volumes of hRBCs. The first injection of 500 µl hRBCs is administered on day 5 postsporozoite challenge, 1 day before exoerythrocytic merozoites start emerging from the liver. To prevent overloading the animals with erythrocytes, this is accompanied by a 200 µl blood draw. Human RBC counts are quantified daily by FACS analysis using a CD235ab antibody, as described before (11). The following day, day 6 postinfection, mice are bled 200 µl and injected 700 µl hRBCs i.p. Following this procedure, already on day 7 the percentage of hRBCs reaches 20–30%, thereby providing a large pool of target cells for the emerging parasites. By day 8, the percentage of hRBCs reaches 50% and in order to keep the percentage stable between 50 and 70% mice are injected daily with 300–700 µl hRBCs. Higher percentages are achievable however this does not increase the success rate of transition, but can increase mortality due to elevated hematocrit. The described protocol is depicted in Figure 2. This protocol allows the maintenance of a stable *P. falciparum* blood stage infection with increasing parasitemia over time.

## Inhibition of Blood Stage Infection by an Antibody Targeting RH5

To assess the utility of our model, we measured for the first time the *in vivo* efficacy of two anti-RH5 human monoclonal antibodies on the transition and establishment of blood stage infection.

**Figure 3:**
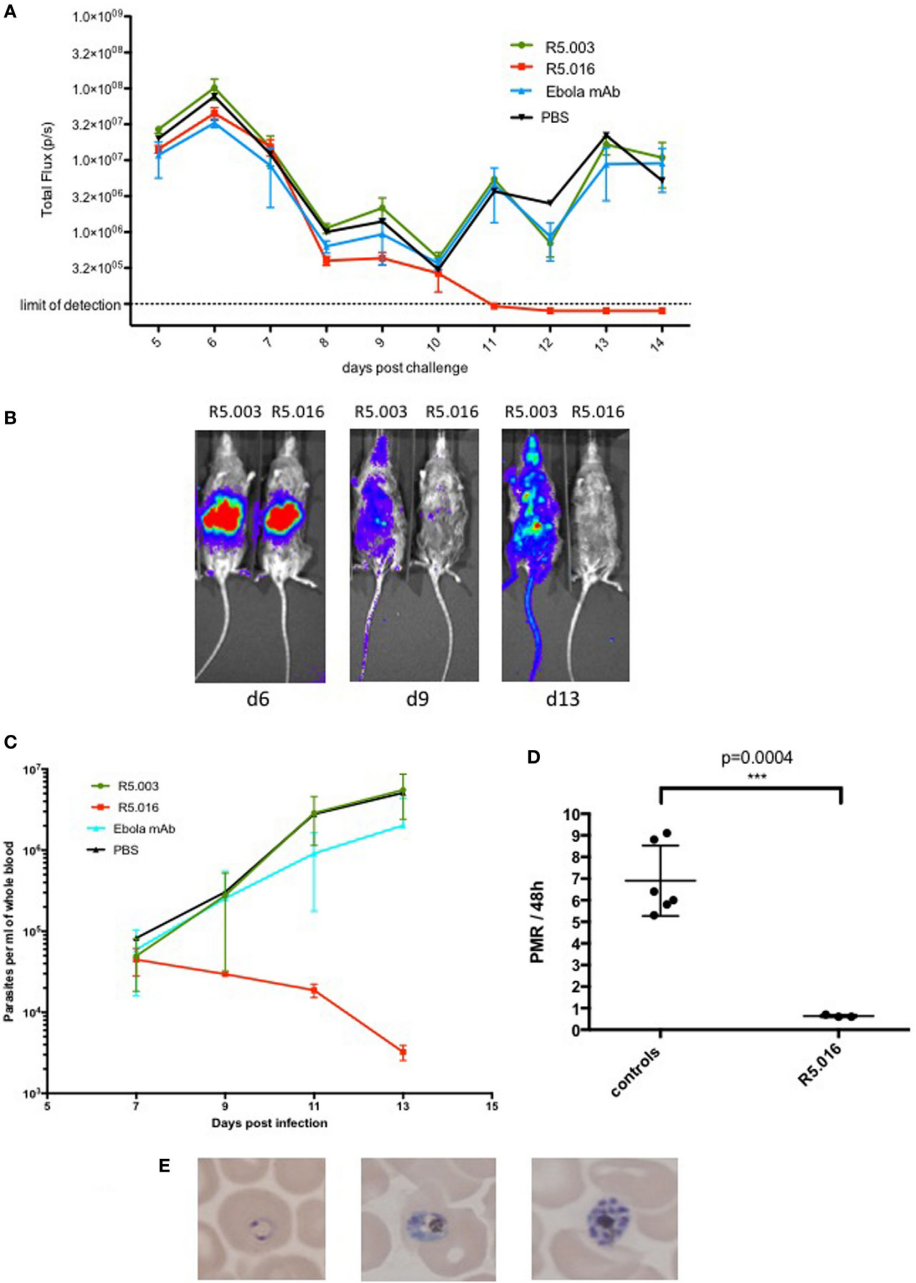
Inhibition of *Plasmodium falciparum* blood stage infection by anti-reticulocyte-binding protein homolog 5 (anti-RH5) antibody. 10 mice were challenged with *P. falciparum* NF54 GFP-Luc infected mosquitoes (*n* = 50 per mouse; 20 min). Blood humanization and passive transfer of RH5 and control monoclonal antibodies (mAbs) was achieved using the protocol as depicted in Figure 2B. **(A)** Mice were imaged daily by IVIS starting 5 days postinfection. They were intraperitoneally injected with 100 μl of Rediject d-luciferin (Perkin Elmer) and imaged after 5 min for a 5-min exposure. Liver and blood-stage burden was assessed by placing an identical region of interest around each mouse and measuring total flux in pixels/second (p/s). The liver-derived IVIS signal peaks on day 6 postinfection and declines thereafter until day 10 due to the transition of the parasite from liver to blood stage infection. After day 10, the blood stage infection increases in all mice except for the ones treated with R5.016. In these mice, the infection decreases further and is undetectable by IVIS by day 11 postinfection. The mean ± SD is shown for each treatment group. **(B)** Representative IVIS images of R5.003 and R5.016 treated animals from days 6, 9, and 13. The liver-derived IVIS signal on day 6 is comparable for both mice. On days 9 and 13, the mouse treated with R5.003 shows a strong IVIS signal distributed throughout the body due to the increasing parasitemia, whereas the mouse treated with R5.016 shows only a very weak signal on day 9 and no signal is detectable on day 13, indicating that blood stage infection was potently inhibited. **(C)** *P. falciparum* parasites per ml of whole blood measured by quantitative reverse transcription (qRT-PCR) targeting *P. falciparum* 18S RNA as described previously (22) showing a dramatic decrease of infection levels only in the mice treated with R5.016. The mean ± SD is shown for each treatment group. **(D)** The parasite multiplication rate per 48 h was calculated for days 9–11 from the qRT-PCR data shown in Figure 3C. Controls refer to the mice treated with R5.003, Ebola mAb, or PBS. One control mouse showed low parasitemia on day 9 but reached levels comparable to the other mice on day 11, therefore the PMR is not comparable to the other mice and this data had to be excluded from the analysis. Each dot represents one mouse and the mean ± SD is shown. **(E)** Giemsa stained thin blood smears were analyzed daily by microscopy. Starting on day 8 postinfection, different developmental stages of asexual parasites could be detected in all animals except for the ones who received R5.016, where no parasites could be detected by microscopy at any time point.

To this end, we infected 10 FRGN huHep mice by *P. falciparum* NF54 GFP-Luc infected mosquito bites (*n* = 50 mosquitoes per mouse; 20 min). Starting 5 days postinfection, mice were imaged daily by IVIS to measure the liver infection level. Based on initial liver stage parasite burden on day 5, the mice were randomized into four groups and received an i.p. injection of vehicle control (PBS, *n* = 1), antibody control (Ebola mAb EBL040, *n* = 3) (Rijal P. et al., in preparation), or anti-RH5 mAb R5.003 (*n* = 3) or R5.016 (*n* = 3) at a dose of 100 mg/kg. *In vitro* assays of growth inhibition activity (GIA) have shown R5.016 is a potent inhibitor of parasite growth, whereas R5.003 does not show any GIA (Alanine et al., in preparation). Confirming these results *in vivo* is of great importance, since it remains highly debated as to whether vaccines or antibodies prioritized on the basis of *in vitro* GIA would subsequently confer *in vivo* efficacy (15). Following our protocol as described above and outlined in Figure 2B, all mice showed similar repopulation levels with hRBCs ranging from 20 to 35% on day 7 and steadily increasing afterward (Figure 2C). Blood stage parasitemia was detectable by quantitative reverse transcription PCR (qRT-PCR) starting on day 7 (Figure 3C), and by IVIS (Figure 3A) and microscopy of thin blood smears (Figure 3E) on day 8. The serum antibody concentrations were similar for all treatment groups throughout the experiment (Figure S1 in Supplementary Material). In animals passively transferred with R5.003 (GIA-negative mAb) the parasitemia increased over time in a manner indistinguishable from the vehicle or antibody control mice. In contrast, the mice passively transferred with R5.016 (GIA-positive mAb) showed no IVIS signal on day 13 postinfection (Figures 3A,B) and no detectable parasites by microscopy of thin blood smears. The infection level by qRT-PCR remained above the limit of detection of 20 parasites/ml for all mice during the 13-day observation period, but a dramatic decrease was seen in the mice passively transferred with R5.016 (Figure 3C).

In addition, we calculated the parasite multiplication rates (PMRs) per 48 h between days 9 and 11 for the mice treated with R5.003, Ebola mAb, or PBS, combined as the control group, and the mice passively transferred with R5.016 (Figure 3D). The PMR per 48 h for the control group is approximately 7-fold, which is similar to the kinetics seen in CHMI studies, where the PMR was shown to be about 10-fold per 48 h (16). The slight difference in PMR between our murine model and CHMI might be due to the fact that in the murine model not all RBCs are of human origin. The mice passively transferred with R5.016 show a PMR of 0.5-fold, indicating that the parasites cannot multiply, most likely due to the invasion-inhibitory effect of R5.016. These passive transfer results confirm that *in vivo* efficacy of the antibodies tested here aligns with their ability to show GIA *in vitro*, and underline the utility of our liver stage/blood stage model to test anti-malarial blood stage interventions.

It has been shown previously that human monocytes act synergistically with antibodies directed against the merozoite surface (23). Since no human monocytes are present in our mouse model and mouse monocytes have been depleted by CloLip treatment, the inhibitory effect of the anti-RH5 mAb R0.016 cannot be attributed to monocyte activity, but likely to direct inhibition of merozoite entry into the RBC. It may be speculated that the presence of human monocytes might increase this inhibitory effect even further.

## Conclusion

In conclusion, we show the establishment of a *in vivo* FRGN huHep/hRBC model to study *P. falciparum* liver stages, the transition to blood stage infection and the development of blood stage parasitemia. As shown by inhibition studies using a mAb targeting RH5, this combined model will be useful to study the effect of novel therapeutics on the different life cycle stages of human *Plasmodium* parasites as well as stage transitions *in vivo*. Because both liver and blood stage infection can be measured separately by intravital imaging, this model will allow us to distinguish the effects on either stages and determine the combined efficacy on the human host cycle of malaria *in vivo*.

Additionally, this improved mouse model will be of great value for the recovery of progeny from genetic crosses. Genetic crosses between phenotypically distinct parasite strains allow the identification of genes controlling drug resistance and other key phenotypes. Previously, *P. falciparum* genetic crosses had to be carried out in splenectomized chimpanzees, but it was recently reported that recombinant progeny can also be recovered from FRG huHep mice that had been injected with hRBCs (24). Parasitemia is low though (typically <0.1%) so that the recovered parasites have to be expanded *ex vivo* before initiating cloning. This may bias the results toward parasites that can replicate better in culture conditions. With our improved protocol which leads to 20–30% hRBCs on day 7 postinfection, when transition from liver to blood stage occurs, higher parasitemias could potentially be achieved, leading to the recovery of a larger population of progeny. Recovery of more clones would lead to a wider variation of progeny and cloning could be initiated directly out of the mouse.

Although this mouse model provides the opportunity to study most aspects of *P. falciparum* infection as well as the effects of novel drug and vaccine candidates, it does require an experienced researcher in order to reproducibly achieve high levels of hRBCs as the volumes of hRBCs injected after day 6 have to be adjusted on a day-to-day basis. To circumvent these issues, the ideal *in vivo* model for malaria research would be a mouse which intrinsically promotes human erythropoiesis. A first step in this direction is the DRAG (HLA-DR4.RagKO.IL2RγcKO.NOD) mouse model, an immune deficient mouse expressing human HLA class II genes (25). These mice can be reconstituted with human hepatocytes, Kupffer cells, liver endothelial cells, and erythrocytes by infusing HLA-matched HSC’s (26). Although the resulting liver (only 0.023 versus 90% for FRG mice) (27) and blood (0.2–1%) humanization was extremely low, injected sporozoites were still able to infect human hepatocytes, leading to a low, but detectable blood stage infection after 10–28 days (3–5 parasites/μl of blood) (26). These low repopulation efficiencies limit the use of DRAG mice, nevertheless raise hope that in the future a human liver chimeric mouse will be developed that additionally promotes human erythropoiesis. Until this is the case, the model we present here will be an extremely useful tool for the *in vivo* study of human *Plasmodium* parasites and the evaluation of novel antimalaria drug and vaccine candidates.

## Ethics Statement

This study was carried out in accordance with the recommendations of the NIH Office of Laboratory Animal Welfare standards (OLAW welfare assurance # A3640-01). The protocol was approved by the Center for Infectious Disease Research Institutional Animal Care and Use Committee (IACUC) under protocol SK-16.

## Author Contributions

LF, CS, NM, DA, EF, RS, BS, and ZB carried out laboratory work and collected and analyzed data. LF and CS drafted the manuscript. NC, MF, WB, and TN produced gametocytes and sporozoite-infected mosquitoes. DA and SD provided the RH5 and Ebola mAbs. JB and EW provided FRGN huHep mice. SM, SD, SM, and SK analyzed data, supervised the work, and contributed to discussion. All authors read and edited the final manuscript.

## Conflict of Interest Statement

JB and EW work for Yecuris Corp., the company that sells FRGN huHep mice. All other authors declare that the research was conducted in the absence of any commercial or financial relationships that could be construed as a potential conflict of interest.

## Supplementary Material

Figure S1 The amount of hIgG1 present in the mouse serum was measured by standardized ELISA, as previously described (28, 29), except that the plates were coated with full-length reticulocyte-binding protein homolog protein expressed in *Drosophila* S2 cells. All mice show similar antibody levels throughout the experiment. Each sample was measured as quadruplicate and the mean ± SD is plotted for each mouse.
