# Supplementary figures and images for: Plasmodium falciparum Liver Stage Infection and Transition to Stable Blood Stage Infection in Liver-Humanized and Blood-Humanized FRGN KO Mice Enables Testing of Blood Stage Inhibitory Antibodies (Reticulocyte-Binding Protein Homolog 5) In Vivo
Source: Front Immunol. 2018 Mar 14;9:524. doi: 10.3389/fimmu.2018.00524 (PMC5861195; doi:10.3389/fimmu.2018.00524)

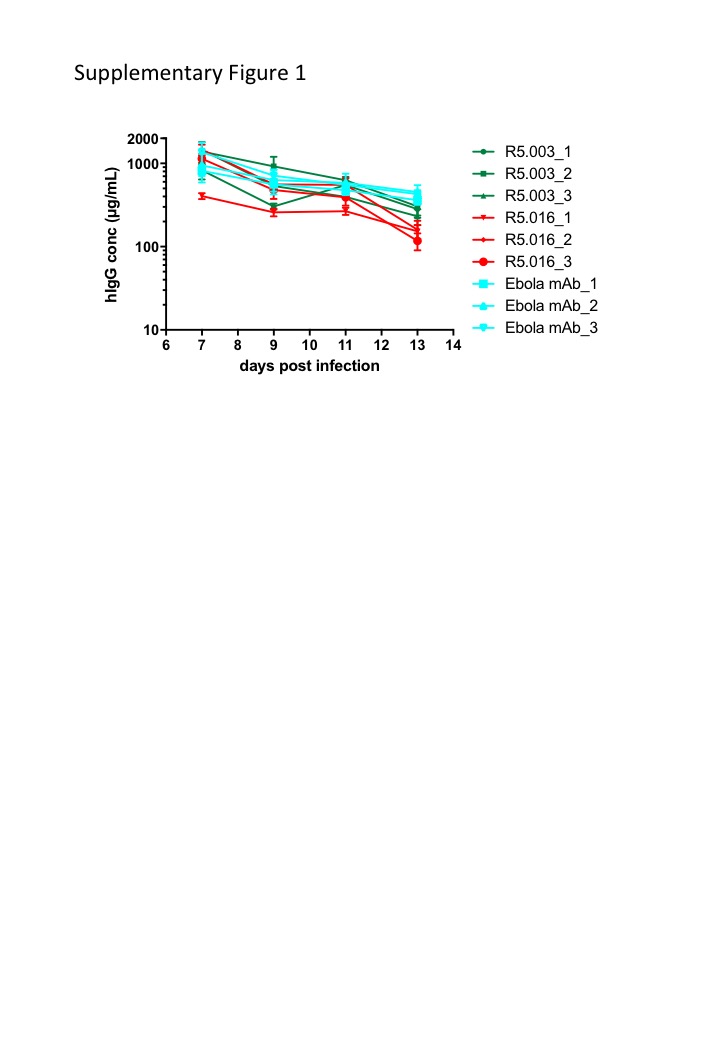

Supplement: Figure S1 — The amount of hIgG1 present in the mouse serum was measured by standardized ELISA, as previously described (28, 29), except that the plates were coated with full-length reticulocyte-binding protein homolog protein expressed in Drosophila S2 cells. All mice show similar antibody levels throughout the experiment. Each sample was measured as quadruplicate and the mean ± SD is plotted for each mouse. [file image_1.jpeg]
